# The Association of Per- and Polyfluoroalkyl Substances Serum Levels and Allostatic Load by Country of Birth and the Length of Time in the United States

**DOI:** 10.3390/ijerph19159438

**Published:** 2022-08-01

**Authors:** Tahir Bashir, Fafanyo Asiseh, Kenrett Jefferson-Moore, Emmanuel Obeng-Gyasi

**Affiliations:** 1 Department of Built Environment, North Carolina Agricultural and Technical State University, Greensboro, NC 27411, USA; tmbashir@aggies.ncat.edu; 2 Environmental Health and Disease Laboratory, North Carolina Agricultural and Technical State University, Greensboro, NC 27411, USA; 3 Department of Economics, Deese College of Business and Economics, North Carolina Agricultural and Technical State University, Greensboro, NC 27411, USA; fasiseh@ncat.edu; 4 Department of Agribusiness, Applied Economics and Agriscience Education, North Carolina Agricultural and Technical State University, Greensboro, NC 27411, USA; jykenret@ncat.edu

**Keywords:** PFAS, allostatic load, country of birth, length of time in the U.S.

## Abstract

Objectives: The aim of this study was to examine the association of per- and polyfluoroalkyl (PFAS) concentrations and allostatic load (AL) by the county of birth and the length of time in the United States of America (U.S.), in a representative sample of U.S. adults. Methods: Data from the 2007–2014 National Health and Nutrition Examination Survey (NHANES) were used in this cross-sectional study on the U.S. adults aged 20 and older. The analysis was stratified by the length of time in the U.S. and by the county of birth. In all, the sample contained those who were US-born (*n* = 10,264), Mexico-born (*n* = 4018), other Spanish speaking country-born (*n* = 2989), and other not–Hispanic speaking country-born (*n* = 3911). Poisson models were used to assess the differences in AL and PFAS levels depending on country of birth and length of time in the U.S. Results: Estimates indicated that those born in Other non–Spanish speaking counties had the highest PFAS levels among the country of birth category in the database. Regarding length of time in the U.S., those born in Mexico had low PFAS levels when their length of time in the U.S. was short. The Mexico-born category presented the most at-risk high serum PFAS levels, with AL levels increasing by length of time in the U.S. (*p*-value < 0.001). Conclusion: This study found that PFAS concentrations increased by the length of time residing in the U.S. Those born in other non–Hispanic counties had the highest PFAS levels among all the categories. In general, AL and PFAS levels are mostly associated with the length of time in the U.S., with foreign-born individuals having increased levels of both the longer they stay.

## 1. Introduction

For more than 50 years, PFAS have been produced and used for consumer products, such as leather and fabric coatings, paper coatings, firefighting foams, and stain repellents [1,2]. Many of the long-chain PFAS bioaccumulate (as opposed to biomagnify) in the human body but not enough of the short-chain PFAS have been studied to rule out their bioaccumulation potential. It is thought that they are less likely to bioaccumulate, but this has not been empirically demonstrated for all that can be classified as short-chain PFAS [3]. Before 2002, most studies of PFAS in the United States of America (U.S.) focused on Perfluorooctanoic acid (PFOA) and perfluorooctane sulfonic acid (PFOS), leading to discontinued production. Nevertheless, these legacy chemical compounds continue in the environment and are still produced and manufactured, utilized (i.e., put PFAS into service for profit), and used (i.e., benefit from PFAS, such as paper packaging, stain resistant coatings, furniture, cosmetics, household cleaners, etc.) worldwide [4,5].

Human exposure to PFAS can occur in occupational settings [6] via cutaneous contact and inhalation. Non–occupational sources such as contaminated drinking water, and seafood (especially fish), are additional sources of exposure. The vast majority of PFAS are highly persistent and have long half-lives in the environment and humans. For instance, two of the most well-studied PFAS, PFOS and PFOA, have half-lives in human blood of roughly between two and five years [2,7,8]. In all, PFAS exposure comes from various sources, with demographic factors and lifestyle sometimes predicting the exposure risk [9,10].

PFAS are usually detected in wildlife, the environment, and humans, making them substances of global concern. The Stockholm Convention on Persistent Organic Pollutants (POPs), governed by the United Nations Environment Program (UNEP), is an international agreement to protect human health and the environment from unfavorable impacts caused by POPs. The convention listed PFOS and its salts and PFOA and its salts. This was so because they have effects on human wellbeing, with scientific evidence suggesting significant impacts on human health. Owing to these health effects and public worry of these chemical compounds, policies have been proposed to mitigate the manufacture and use of PFAS [11,12]. A global approach to PFAS is critical since people move freely worldwide.

Immigration to the U.S. has significantly increased in the last few decades compared to any other time in history. Many of these immigrants may be from countries where they may be at greater risk of exposure to contaminants from the environment [13,14]. In general, immigrants in the U.S. encounter various challenges, which can result in exposure to various toxic chemicals in a community. In addition, the effects of immigration and assimilation can contribute to stress and subsequent adverse health outcomes [15,16,17,18].

Allostasis is how individuals maintain physiological balance by altering parameters within the body and matching them to environmental demands. Allostasis is similar to homeostasis but is different in that homeostasis defines health as a state in which all physiological parameters operate within non-changing setpoints [19,20]. With allostasis, individuals can appropriately respond to challenges, but if the challenges are continuous and do not turn off, then the body, rather than going to a lower set point, adapts at the higher set point. When the setpoint changes, it is called allostatic load. Allostatic load (AL), an index of chronic physiological stress, is the biological penalty of stress. AL is based on the assumption that repetitive activation of the hypothalamus, pituitary gland, and adrenal (HPA axis) affects multiple organ systems, such as the cardiovascular system [15,19,20,21,22]. In a database such as NHANES, AL can be measured by combining markers from the cardiovascular, metabolic and inflammatory system.

This study aimed to examine the associations between AL and select PFAS by country of birth and length of time in the U.S. We hypothesized that country of birth and length of time in the U.S. would be significant factors in exposure to PFAS and AL levels. We examined variations by county of birth, demographic groups, income, and education to further explore these relationships.

## 2. Materials and Methods

### 2.1. Cohort and Study Design

This study used data from National Health and Nutrition Examination Survey (NHANES) 2007–2014 among adults aged 20 and over. The NHANES is a representative sample of noninstitutionalized individuals living in the 50 states of the U.S. and the district of Columbia. The data, collected by the U.S. Centers for Disease Control and Prevention (CDC), contains multi-year, stratified, multistage, and clustered samples, with data available in two-year cycles. The data for this analysis represent the U.S population for four cycles 2007–2008, 2009–2010, 2011–2012, and 2013–2014. NHANES interviewed selected participants, and they also completed a physical examination. Blood samples were drawn from participants and were sent to a laboratory for analysis. More descriptions and details on the study and procedures and processes involved are found on the CDC’s NHANES website [23].

Weighted (i.e., weighted data in NHANES is used to make the sample estimates reflect how many people in the U.S. population one individual represents among civilian noninstitutionalized residents) [24] and pooled (i.e., the combination of NHANES data form 2007 to 2014) data were used for this analysis to assess the association between the different PFAS concentrations and AL levels. This was assessed by county of birth and the length of time in the U.S.

### 2.2. Allostatic Load Levels Determination

In determining the AL levels, the cut-off points for selected biomarkers were determined based on the distribution of the variables within the database [15]. We explored cardiovascular, metabolic, and inflammatory markers, including systolic blood pressure (SBP), diastolic blood pressure (DBP), total cholesterol (TC), high–density lipoprotein (HDL) cholesterol, triglyceride (TG), glycosylated hemoglobin (HbA1c), Body mass index (BMI), albumin (Alb), creatinine clearance (CRCL), and C–reactive protein (CRP). Individual biomarkers were assigned to a binary value of 1 if the biomarker was among the high–risk category, that is, the top 25% in the database, and a value of 0 if not for all markers apart from albumin, creatinine clearance, and HDL cholesterol for which the bottom 25% were the high risk. A total AL score was calculated out of 10, with an AL greater than or equal to 3 considered a high AL.

### 2.3. Study Variables and Measures

We used these variables as predictors: Length of time in the U.S. which had nine categories (1) Less than 1 year, (2) 1 year, less than 5 years or 1–4, (3) 5 years, less than 10 years or 5–9, (4) 10 years, less than 15 years or 10–14, (5) 15 year., less than 20 years or 15–19, (6) 20 years, less than 30 years or 20–29, (7) 30 years, less than 40 years or 30–39, (8) 40 years, less than 50 years or 40–49, and (9) 50 years or more. Education level, specifically for adults 20 years and older, had five categories: (1) Less than 9th grade, (2) 9th to 11th grade (Includes 12th grade with no diploma), (3) High school graduate/GED or equivalent, (4) Some college or Associate of Arts (AA) degree, and (5) College graduate or above. Country of birth in this database four categories: (A) Born in 50 US States or Washington, DC, (B) Born in Mexico, (C) Born in Other Spanish Speaking County, and finally, (D) Born in other Non-Spanish Speaking Country. Health behavior variables, including smoking. alcohol consumption behaviors, and physical activity were also explored as covariate variables that could influence the outcomes.

PFAS concentrations were measured from participants’ samples at the medical examination center with a lower limit of detection (LLOD) of 0.07 μg/L. In the lab analysis, serum concentrations below the LLOD were replaced with the LLOD divided by the square root of 2.

### 2.4. Statistical Analysis

Descriptive statistical analysis was used to generate the mean, and standard deviation with graphs (boxplots) was used to present the variabilities between groups. In our analysis, a *p*-value ≤ 0.05 was considered significant. Poisson models and Pearson correlation coefficient tests were used to assess the associations between variables on interest in the study. A programming language for statistical computing and graphics named R in RStudio (R Foundation for Statistical Computing, Vienna, Austria) was used for these analyses. Confounding variables were identified based on proportion of selected variables and primary assessment of the variables’ association with length of time in the U.S., country of birth, PFAS and AL. Then, adjusted models for age, education, and gender were performed.

## 3. Results

In all, the sample contained those who were US-born (*n* = 10,264), Mexico-born (*n* = 4018), other Spanish speaking country-born (*n* = 2989), and other not-Hispanic speaking country-born (*n* = 3911). The sample mean and standard deviation of AL scores are presented in Table 1 by the length of time in the U.S. and country of birth. Among the immigrants to the U.S. The AL means for participants born in Mexico who had been in the U.S. for less than one year to over 50 years ranged between 2.5 to 3.6, with standard deviations (SD) ranging between 1.2 to 1.6. For the immigrants born in other non-Spanish speaking counties whose time in the U.S went from less than one year to greater than fifty years, the mean AL values were between 2.2 and 3.3. Those who identified their country of birth as ‘other Spanish speaking country’ had mean AL levels, which ranged between 2.5 and 3.6. Finally, among the non-immigrants (those who indicated they were born in the U.S.), participants had means AL levels between 2.7 to 3.5. Overall, the trends suggest that AL tends to increase by the length of time in the U.S.

Table 1 shows SD and means for the participants according to country of birth and the length of time the U.S. In general, as length of time in the U.S. increased, AL levels increased as well. The participants’ who had been in the U.S for more than 50 years had the highest AL levels.

Poisson model analysis was used to examine the statistical relationship between selected variables (Table 2). The participants’ who had been in the U.S for more than 50 years had statistically significantly (*p*-value < 0.001) higher AL for all countries of birth categories.

**Figure 1:**
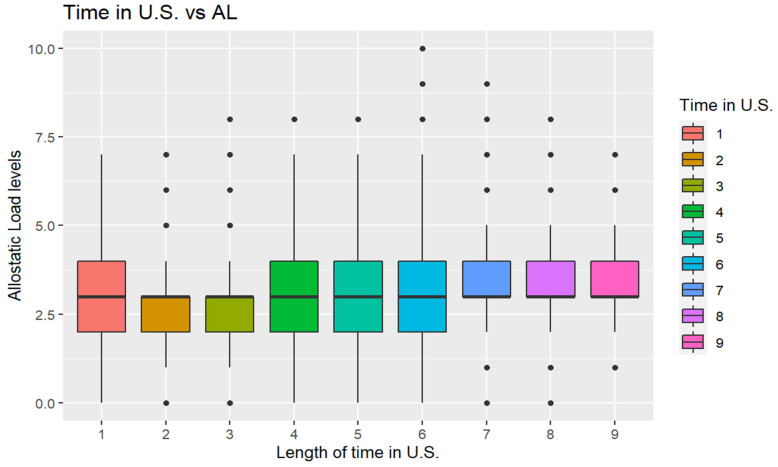
Boxplot (box and whisker plot) shows AL concentrations by length of time in U.S. Note: The length of time in U.S. (1) Less than 1 year (1 year <), (2) 1 year, less than 5 years (1–4), (3) 5 years, less than 10 years (5–9), (4) 10 years, less than 15 (10–14), (5) 15 years, less than 20 (15–19), (6) 20 years, less than 30 years (20–29), (7) 30 years, less than 40 years (30–39), (8) 40 years, less than 50 years (40–49), and (9) 50 years or more (≥50).

We explored AL level by the length of time in the U.S. Figure 1 reveals that AL had similar levels for the participants throughout the time they had been in the U.S., but those who had been in the U.S. for less than 1 year (<1 year), between 10 years and 15 years (10–14), between 15 years and 20 years (15–19), and between 20 and 30 years (20–29) had AL levels which evenly spanned the distribution of the boxplot around the median.

Examining the distribution by length of time, those who had been in the U.S. for less than one year, between one year and five years (1–4), and between five and 10 years (5–9) had AL levels mostly below the median. Those who had been in the U.S. between 30 and 40 years (30–39), between 40 and 50 years (40–49), and for more than 50 years (≥50) had AL levels above the median.

Table 3 presents sample means of PFAS concentrations by country of birth. Notice that PFOS was the highest detected for all groups. The mean concentration of PFOS for other non-Spanish-speaking countries of birth was significantly higher than the other groups, followed by those born in other Spanish speaking countries.

Table 4 reveals that PFAS concentrations were at different levels by the length of time in the U.S. For instance, PFDE tended to increase by the time in the U.S. till the 19th year. From the 20th year on, it stayed steady with slight changes.

**Figure 2:**
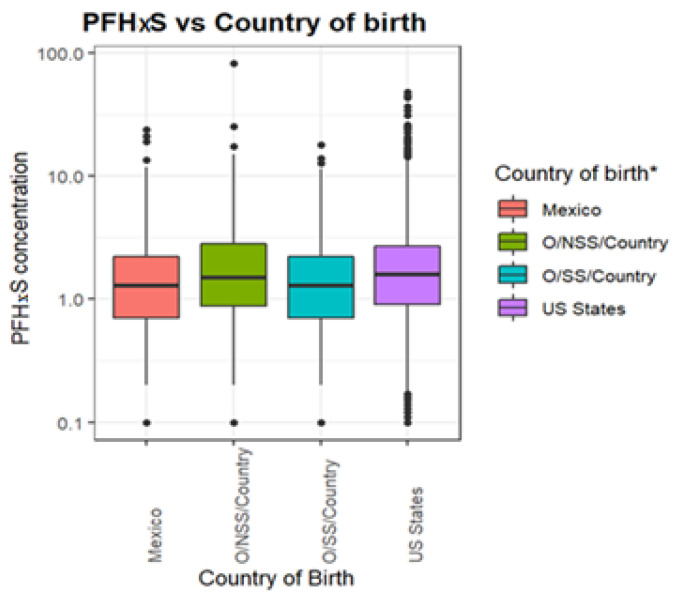
PFHxS (ng/mL) levels by county of birth. * Note: Mexico is Mexican born, US States is U.S born, Other Spanish Speaking County is O/SS/Country, Other Non-Spanish Speaking Country is O/NSS/Country.

Figure 2 shows that PFAS serum levels were similar among the participants with a slight difference in people who were born in Mexico who tended to have lower values.

To evaluate if the length of time in the U.S. and county of birth has an association with AL levels and PFAS serum concentrations, we employed Pearson correlation analysis (Table 5). The Pearson correlation analysis is presented in Table 5 below, examining the relationship between selected variables. AL had a very weak negative association with PFOS, country of birth, and education level, with r values of –0.001, –0.081, and –0.070, respectively. It also had a very weak positive correlation with length of time in U.S. with r values of 0.009 and 0.114, correspondingly. We used PFOS to represent PFAS because nearly all participants had considerably high detected levels of it. The association of PFOS with education level had very weak correlations with an r-value of –0.022. PFOS also had a very weak association with country of birth with an r-value of 0.054. Its association with length of time in the U.S. was not strong, with an r-value of 0.

Mean levels of PFOS and AL by length of time in the U.S. are explored in Table 6. Results indicated that those who had been in the US for more than 50 years had the highest mean PFOS and AL levels.

In adjusted Poisson models, there was a significant difference in AL levels for those who had been in the U.S. for more than fifty years as compared to newcomers among all groups (Table 7). AL levels increased by the time in the U.S. For instance, those who had been in the U.S. 40–49 years had, on average, a 3.3% higher AL and those who had been in the U.S. more than 50 years had on average 3.4% higher AL levels compared to those who had been in the U.S. less than one year (newcomers).

## 4. Discussion

In this study, we investigated the relationships between exposure to PFAS and AL by country of birth and the length of time in the U.S. An increased length of time in the U.S. was associated with the rising AL levels. This finding is consistent with research conducted by Doamekpor and colleagues, which indicated that increasing AL was associated with increased length of residency in the U.S. [17]. Immigrants born in non–Spanish speaking countries had high AL levels, with individuals who were born in Mexico having considerable PFAS exposure and AL.

Our study revealed that those over 50 years old had high levels of AL among all categories of countries of birth in this study. The finding of high AL matched the length of time in the U.S. and indicated that the longer one had been in the U.S., the higher their AL levels became. These results are consistent with a study by Peek and colleagues, which indicated that foreign-born individuals who had recently arrived in the U.S. had relatively better health on arrival, but their health declined over time, with AL increasing concurrently [25]. The stressors associated with immigration, assimilation, and adaptation to a new country and culture may explain this finding.

Our study result indicated that PFAS and AL levels elevated by the length of time in the U.S. potentially indicates that emerging environmental contaminants like PFAS may potentially pose a differential geographic exposure pattern compared to traditional contaminants like Lead. Lead tends to exist in higher concentrations in low- and middle-income countries (LMIC) as compared to high-income countries (HIC) [14].

The individuals who were born in Mexico were the most at risk of elevated AL levels by the length of time in the U.S., which was consistent with a study by Kaestner and colleagues, which indicated that the relationship between AL and the participants born in Mexico was statistically significant (*p*-value < 0.001). Specifically, Mexico-born participants were healthier upon arrival in the U.S., and their health diminished over time [26]. This is also compatible with the study by Doamekpor and colleagues, which indicated that the people born in countries other than the U.S. had lower AL levels, and their AL increased over time [17].

Limitations: Trends in this study assessed by the length of time have limitations as the study design was cross-sectional. A longitudinal study is necessary to reach definitive conclusions.

## 5. Conclusions

Our study revealed that there is a significant association between self-reported length of time in the U.S. and PFAS and AL levels. These levels were highest for those reporting living in the U.S. for an extended period. These results, along with prior related studies, suggest newcomer populations are the subject of concern for elevated PFAS and AL.

## Figures and Tables

**Table 1:** AL level by country of birth and length of time in U.S.

Time in U.S. *	Country of Birth **	Mean	SD
(<1 yr)	Mexico	2.848	1.642
(<1 yr)	O/NSS/Country	2.935	1.504
(<1 yr)	O/SS/Country	3.227	1.378
(<1 yr)	U.S. States	2.729	1.115
(1–4)	Mexico	2.458	1.181
(1–4)	O/NSS/Country	2.227	1.275
(1–4)	O/SS/Country	2.571	1.388
(1–4)	U.S. States	2.958	1.262
(5–9)	Mexico	2.62	1.416
(5–9)	O/NSS/Country	2.414	1.233
(5–9)	O/SS/Country	2.504	1.412
(5–9)	U.S. States	2.97	1.148
(10–14)	Mexico	2.992	1.465
(10–14)	O/NSS/Country	2.76	1.197
(10–14)	O/SS/Country	2.835	1.183
(10–14)	U.S. States	3.165	1.209
(15–19)	Mexico	2.977	1.402
(15–19)	O/NSS/Country	2.774	1.196
(15–19)	O/SS/Country	2.787	1.441
(15–19)	U.S. States	3.164	1.192
(20–29)	Mexico	3.119	1.366
(20–29)	O/NSS/Country	2.81	1.335
(20–29)	O/SS/Country	3.185	1.403
(20–29)	U.S. States	3.306	1.389
(30–39)	Mexico	3.324	1.408
(30–39)	O/NSS/Country	3.174	1.393
(30–39)	O/SS/Country	3.583	1.354
(30–39)	U.S. States	3.493	1.237
(40–49)	Mexico	3.562	1.492
(40–49)	O/NSS/Country	3.188	1.244
(40–49)	O/SS/Country	3.459	1.302
(40–49)	U.S. States	3.649	1.17
(≥50)	Mexico	3.596	1.05
(≥50)	O/NSS/Country	3.329	1.236
(≥50)	O/SS/Country	3.379	1.226
(≥50)	U.S. States	3.623	1.154

* Note: U.S. States = U.S. States born, Mexico = Mexico born, Other Spanish Speaking County = O/SS/Country, Other Non–Spanish Speaking Country = O/NSS/Country. ** Note: The length of time in U.S. (1) Less than 1 year (1 year <), (2) 1 year, less than 5 years (1–4), (3) 5 years, less than 10 years (5–9), (4) 10 years, less than 15 (10–14), (5) 15 years, less than 20 (15–19), (6) 20 years, less than 30 years (20–29), (7)30 years, less than 40 years (30–39), (8) 40 years, less than 50 years (40–49), and (9) 50 years or more (≥50).

**Table 2:** Poisson modes for selected variables with AL.

Time in U.S.	Country of Birth	Coeff	*p*-Value
(<1 yr)	Mexico	1.268	<0.001
(<1 yr)	O/NSS/Country	0.063	0.7
(<1 yr)	O/SS/Country	0.272	0.1
(<1 yr)	US States	0.018	0.9
(1–4)	Mexico	1.026	<0.001
(1–4)	O/NSS/Country	−0.076	0.4
(1–4)	O/SS/Country	0.105	0.38
(1–4)	US States	0.167	0.03
(5–9)	Mexico	0.105	<0.001
(5–9)	O/NSS/Country	0.045	0.64
(5–9)	O/SS/Country	0.004	0.97
(5–9)	US States	0.181	0.03
(10–14)	Mexico	0.050	<0.001
(10–14)	O/NSS/Country	0.040	0.64
(10–14)	O/SS/Country	0.044	0.71
(10–14)	US States	0.142	0.03
(15–19)	Mexico	0.016	<0.001
(15–19)	O/NSS/Country	0.047	0.65
(15–19)	O/SS/Country	−0.008	0.93
(15–19)	US States	0.116	0.12
(20–29)	Mexico	0.030	<0.001
(20–29)	O/NSS/Country	−0.044	0.62
(20–29)	O/SS/Country	0.074	0.42
(20–29)	US States	0.045	0.44
(30–39)	Mexico	0.198	<0.001
(30–39)	O/NSS/Country	−0.086	0.33
(30–39)	O/SS/Country	0.194	0.08
(30–39)	US States	0.058	0.42
(40–49)	Mexico	0.068	<0.001
(40–49)	O/NSS/Country	−0.141	0.24
(40–49)	O/SS/Country	−0.053	0.66
(40–49)	US States	0.002	0.98
(≥50)	Mexico	1.268	<0.001
(≥50)	O/NSS/Country	1.350	<0.001
(≥50)	O/SS/Country	1.688	<0.001
(≥50)	US States	1.259	<0.001

**Table 3:** PFAS mean (ng/mL) and standard deviation by the country of birth.

	* Country of Birth							
	Mexico		O/NSS/Country		O/SS/Country		US States	
PFAS	mean	SD	mean	SD	mean	SD	mean	SD
PFDE	0.147	0.218	0.268	1.143	0.171	0.317	0.154	0.474
PFNA	0.406	0.681	0.545	1.078	0.484	0.871	0.423	1.047
PFOS	3.557	7.998	5.635	15.972	3.962	11.628	3.049	8.9
PFUA	0.106	0.135	0.259	1.083	0.148	0.285	0.128	0.667
PFOA	0.996	1.814	1.15	2.232	1.149	2.044	0.826	2.063
PFHxS	0.621	1.603	0.824	3.271	0.647	1.482	0.722	1.81

* Note: US. States = US States born, Mexico = Mexico born, Other Spanish Speaking County = O/SS/Country, Other Non–Spanish Speaking Country = O/NSS/Country.

**Table 4:** PFAS mean (ng/mL) and standard deviation by the length of time in U.S.

Length of Time in the U.S.																		
	(<1 yr)		(1–4)		(5–9)		(10–14)		(15–19)		(20–29)		(30–39)		(40–49)		(≥50)	
PFAS	mean	SD	mean	SD	mean	SD	mean	SD	mean	SD	mean	SD	mean	SD	mean	SD	mean	SD
PFDE	0.167	0.254	0.175	0.297	0.201	0.592	0.204	0.460	0.198	0.439	0.152	0.519	0.191	0.462	0.152	0.222	0.145	0.244
PFNA	0.318	0.516	0.405	0.749	0.443	0.751	0.452	0.947	0.449	0.870	0.427	1.066	0.446	0.911	0.453	0.994	0.400	0.669
PFOS	2.357	5.551	2.539	7.668	2.361	5.659	2.331	6.038	3.067	7.705	3.317	9.620	3.396	9.598	3.038	7.307	3.699	16.325
PFUA	0.175	0.357	0.179	0.377	0.165	0.323	0.181	0.471	0.166	0.381	0.125	0.718	0.16	0.366	0.132	0.191	0.121	0.207
PFOA	0.691	1.353	0.672	1.375	0.744	1.548	0.656	1.545	0.706	1.388	0.896	2.167	0.759	1.651	0.849	1.865	0.848	1.777
PFHxS	0.304	0.589	0.415	0.946	0.449	0.882	0.514	1.207	0.588	1.269	0.750	1.891	0.798	3.174	0.770	1.781	0.808	1.618

Note: The length of time in U.S. (1) Less than 1 year (1 year <), (2) 1 year, less than 5 years (1–4), (3) 5 years, less than 10 years (5–9), (4) 10 years, less than 15 (10–14), (5) 15 years, less than 20 (15–19), (6) 20 years, less than 30 years (20–29), (7) 30 years, less than 40 years (30–39), (8) 40 years, less than 50 years (40–49), and (9) 50 years or more (≥50).

**Table 5:** Correlation between AL, PFOS (ng/mL), Country of Birth, Length of Time in the U.S., and Education Level.

	AL	PFOS	Country of Birth	Length in U.S.	Education
AL	1.000	−0.001	−0.081	0.114	−0.070
PFOS		1.000	0.054	0.026	−0.022
Country of birth			1.000	−0.171	−0.086
Length in U.S.				1.000	0.039
Education					1.000

**Table 6:** Mean PFOS concentration and AL by length of time in the U.S.

	(<1 yr)	(1–4)	(5–9)	(10–14)	(30–39)	(40–49)	(≥50)
Variable	mean	mean	mean	mean	mean	mean	mean
AL	2.87	2.68	2.72	3.04	3.43	3.53	3.52
PFOS	2.357	2.539	2.361	2.331	3.396	3.038	3.699

**Table 7:** Exp(β) (95% CI) for the adjusted association of length of time in the U.S. with AL.

Length of Time in U.S.	ALL ≥ 20 Years	Men ≥ 20 Years	Women ≥ 20 Years
Time in U.S.			
(1 yr. <) *	0.940 (0.849 to 1.031)	−0.005 (−0.239 to 0.228)	−0.012 (−0.130 to 0.152)
(1–4)	0.894 (0.840 to 0.949)	0.118 (−0.023 to 0.258)	0.110 (−0.014 to 0.300)
(5–9)	0.926 (0.871 to 0.982)	0.114 (−0.010 to 0.239)	0.109 (−0.004 to 0.215)
(10–14)	1.004 (0.961 to 1.048)	0.166 (0.041 to 0.290)	0.105 (0.081 to 0.216)
(15–19)	0.977 (0.937 to 1.018)	0.206 (0.097 to 0.315)	0.200 (0.080 to 0.296)
(20–29)	0.972 (0.928 to 1.017)	0.108 (0.003 to 0.214)	0.101 (0.011 to 0.230)
(30–39)	1.029 (0.977 to 1.082)	0.104 (−0.019 to 0.228)	0.103 (0.001 to 0.201)
(40–49)	1.033 (0.985 to 1.082)	0.112 (−0.037 to 0.260)	0.107 (−0.051 to 0.202)
(≥50)	1.034 (0.969 to 1.099)	1.093 (0.904 to 1.232)	1.005 (0.903 to 1.223)

Models adjusted (Poisson) for education, age and sex. U.S. born, *n* = 10,264 (men and women, ≥20 years old); *n* = 4159 (men, ≥20 years old); *n* = 6105 (women, ≥20 years old). Non-U.S. born, *n* = 10,918 (men and women, ≥20 years old); *n* = 3986 (men, ≥20 years old); *n* = 6932 (women, ≥20 years old). * Reference group. AL, Allostatic Load; CI, confidence interval.

## Data Availability

The NHANES dataset is publicly available online, accessible at cdc.gov/nchs/nhanes/index.htm (accessed on 12 June 2022).
